# Dual medial and anterior approach for excision of extraosseous synovial hip osteochondroma: a case report

**DOI:** 10.1186/s13256-022-03724-w

**Published:** 2022-12-31

**Authors:** Mohamad Issa, Ahmad Naja, Serge Sultanem, Charbel Elias, Said Saghieh

**Affiliations:** Orthopaedic Surgery Department, American University of Beirut Medical Center, Beirut, Lebanon

**Keywords:** Hip osteochondroma, Smith-Petersen approach, Ferguson approach, Case report

## Abstract

**Background:**

Osteochondromas are the most common benign bone tumors occurring near the end of long bones. In this case report, we demonstrate the successful treatment of a proximal femoral osteochondroma in a pediatric patient excised through a dual medial and anterior approach with no hip dislocation.

**Case presentation:**

We present the case of a white Arab 14-year-old boy with chronic hip pain and inability to ambulate. He failed conservative treatment and was referred to us after X-rays revealed two osseous masses. He was diagnosed with an intra-articular hip osteochondroma confirmed on magnetic resonance imaging and computed tomography scan. He was treated surgically with excision using two incisions: Smith-Petersen approach and Ferguson approach.

**Conclusion:**

This case presents the successful resection of a symptomatic pediatric proximal femoral osteochondroma, using dual medial and anterior approaches without the need for hip dislocation. This was optimal for both the safety and accessibility of this unusual condition.

## Background

Osteochondromas are benign osteocartilaginous tumors occurring near the end of long bones, typically forearm, knees, and hips [1]. They are the most common benign bone tumors and account for one-third of all benign bone tumors [2]. Ninety percent of cases present as a solitary lesion; however, multiple lesions can present as part of hereditary multiple exostosis (HME) [2]. Single lesions tend to present in the second and third decades of life, while HME lesions occur during the first decade. Osteochondromas are usually extra-articular lesions and originate from the metaphysis of long bones [1]. These lesions are typically asymptomatic and do not need surgical intervention [3]. However, the increased volume could cause mechanical symptoms including pain, limited range of motion, cosmetic deformity, growth disturbances, and compression of vital neurovascular structures necessitating surgical intervention [3]. The rate of malignant transformation can reach up to 25% in patients with HME compared with less than 1% in solitary lesions [1].

Osteochondromas of the femoral neck are atypical as they represent an intra-articular lesion [4]. However, several studies in the literature have reported the occurrence of these lesions in the proximal femur and acetabulum [4, 5]. They may lead to mechanical restriction of the hip range of motion and damage of the labrum and cartilage. The surgical excision of hip osteochondromas remains challenging for many orthopedic surgeons as they are not commonly encountered in clinical practice [6]. The purpose of this case report is to demonstrate the successful treatment of a proximal femoral osteochondroma in a pediatric patient excised through dual medial and anterior approaches with no hip dislocation.

## Case presentation

A 14-year-old white Arab boy presented with left hip pain of 1-month duration associated with gait imbalance and limping. The patient exhibited a healthy appearance with an appropriate weight (69 kg) for his height (169 cm). His chest and abdominal examination were unremarkable on palpation and auscultation. No lymph node enlargement was identified on neck, axillary, or groin assessment. No back skin lesions or spinal deformities were identified. He demonstrated no neurological deficits with normal cranial nerve assessment. He was able to heel and toe walk with motor power 5/5 in bilateral upper and lower extremities and grossly intact sensation. Peripheral reflexes were all within normal limits. His extremities exhibited normal vascular examination, regular capillary refill, and 3+ pulses in the radial, ulnar, dorsalis pedis, and posterior tibial arteries assessment. The left hip range of motion was unremarkable except for limited internal rotation and pain on adduction and flexion. External rotation of the hip was normal. There was focal tenderness on deep palpation of the medial and anterior aspect of the hip with no palpable lesions. Radiographs of the hip at a peripheral hospital showed two osseous masses around the left femoral neck.

**Fig. 1:**
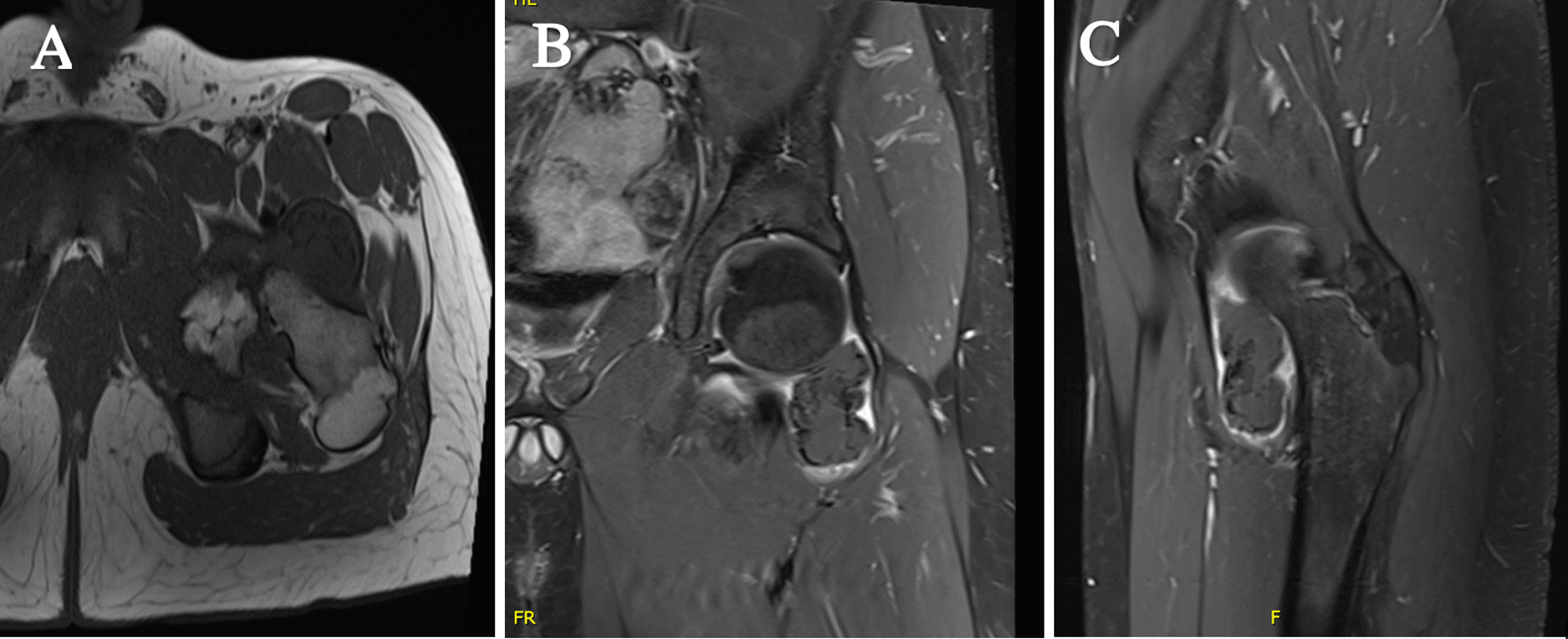
**A** Axial T1, **B** coronal T1, and **C** sagittal T1-weighted MRI scans of the left hip showing the anterolateral lesion with a cartilage cap

**Fig. 2:**
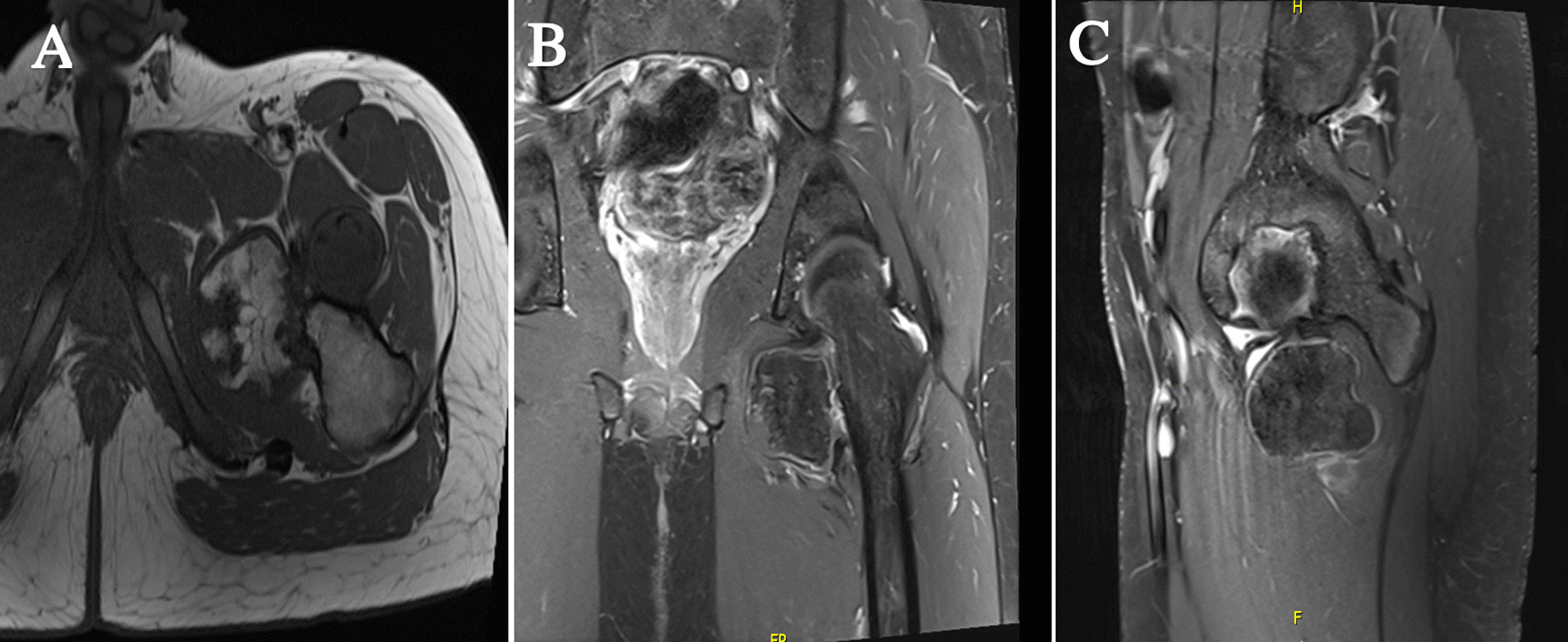
**A** Axial T1, **B** coronal T1, and **C** sagittal T1-weighted MRI scans of the left hip showing the inferomedial lesion with a cartilaginous rim and mature internal ossification

**Fig. 3:**
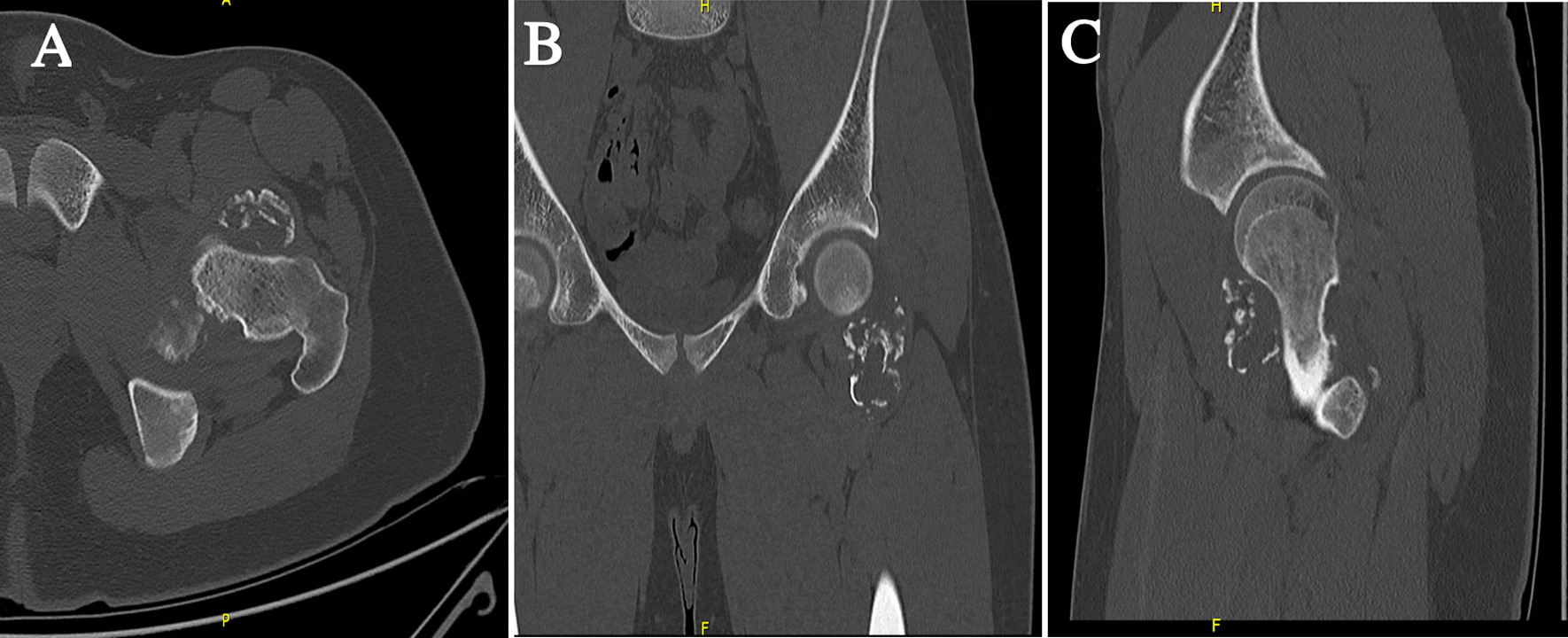
**A** Axial, **B** coronal, and **C** sagittal CT scans of the left hip showing the anterolateral lesion with a cartilage cap

**Fig. 4:**
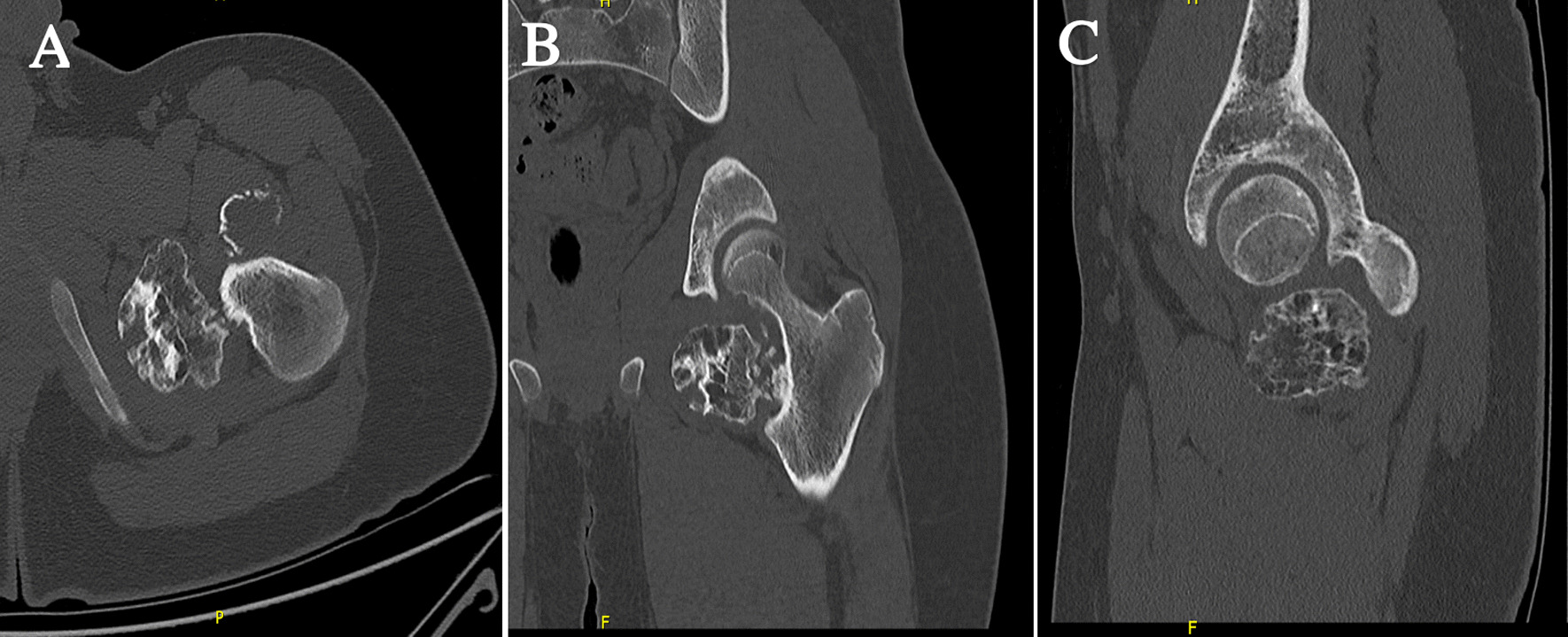
**A** Axial, **B** coronal, and **C** sagittal CT scans of the left hip showing the inferomedial lesion

Magnetic resonance imaging (MRI) of the hip confirmed the two masses as interarticular chondromas with different stages of maturity and no soft tissue extension or malignant degeneration. One lesion on the anterolateral aspect of the left hip joint measuring 4.6 × 3 × 2.5 cm showed signal intensity suggestive of cartilage component and possible partial mineralization (Fig. 1). The second lesion was along the inferomedial aspect of the left hip joint measuring 5.8 × 4 × 4.5 cm showing a cartilaginous rim with mature internal ossification (Fig. 2). A nonenhanced computed tomography (CT) scan of the pelvis and hip joints was further done for evaluation of the matrix component and to rule out communication with the femur (Figs. 3, 4). The CT scan showed more calcification in the inferomedial lesion and no direct bone bridging or communication with the diaphysis of either lesion. Surgical intervention was recommended to relieve the pain and improve the range of motion.

**Fig. 5:**
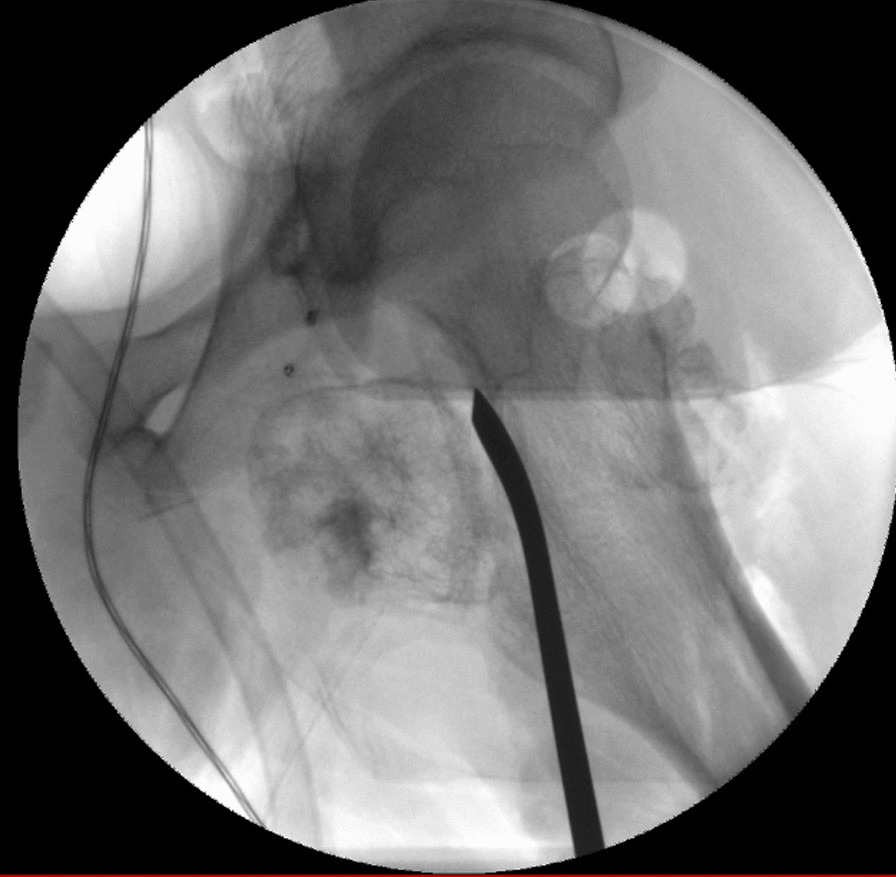
Intraoperative fluoroscopy scan showing the separation of the lesion stalk from the femoral neck using an osteotome

**Fig. 6:**
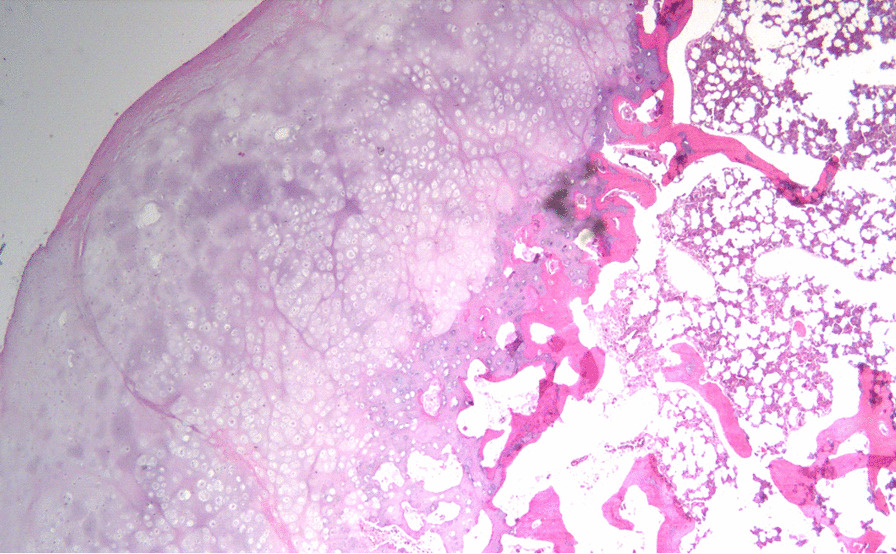
Histopathology slide showing the cartilage cap lined by perichondrium and contiguous with mature bone (hematoxylin and eosin stain)

The patient was scheduled for resection on 29 January 2020. On admission, the patient demonstrated normal vital signs (blood pressure 120/60 mmHg, pulse 86 beats per minute, temperature 36.6 °C, respiratory rate 20 breaths per minute, oxygen saturation 100%). On physical examination, the patient exhibited an antalgic gait with limited hip movement. He had clear lung sound and no murmurs on auscultation of the chest. Abdominal examination was normal on palpation and auscultation. His vascular exam was normal with 3+ pulses and normal capillary refill. Hip examination revealed focal tenderness on palpation of the anterior aspect of the hip with limited range of motion on flexion, adduction, and internal rotation. On neurological examination, all cranial nerves were intact, motor power was 5/5 in all extremities, and his sensation was grossly intact to pinprick test. He also demonstrated normal 2+ peripheral reflexes along with downgoing toes on Babinski test bilaterally. Laboratory studies were unremarkable (complete blood count 15.6 mil /cu.mm, white blood count 8400 /cu.mm, platelet count 228,000 /cu.mm, creatinine 0.9 mg/dL, prothrombin time 12.8 sec, activated partial thromboplastin clotting time 32.8 sec, and normal urine analysis). The surgery was performed under general anesthesia, and a dual incision utilizing both a medial and an anterior approach was deemed necessary for access to both intra-articular lesions. The medial mass was addressed first. A longitudinal incision was made over the palpable tendon of the adductor longus muscle. The interval between the gracilis and adductor longus was identified and developed, followed by the interval between the adductor brevis and adductor magnus muscle. Care was taken to identify the posterior division of the obturator nerve, and the hip capsule was opened proximally uncovering the lesion. The lesion was then dissected free from the surrounding tissues and its stalk separated from the femoral neck using an osteotome with no need for hip dislocation (Fig. 5). The second lesion was accessed using an anterior Smith-Petersen approach to the hip. The interval between the sartorius and tensor fascia lata (TFL) was identified and developed while avoiding injury to the lateral femoral cutaneous nerve. The interval between the rectus femoris and gluteus medius muscles was then identified, and the anterior hip capsule was exposed and opened. The anterolateral lesion was found to be free floating and was extracted from the joint. The two lesions were later confirmed to be osteochondromas based on pathology (Fig. 6).

**Fig. 7:**
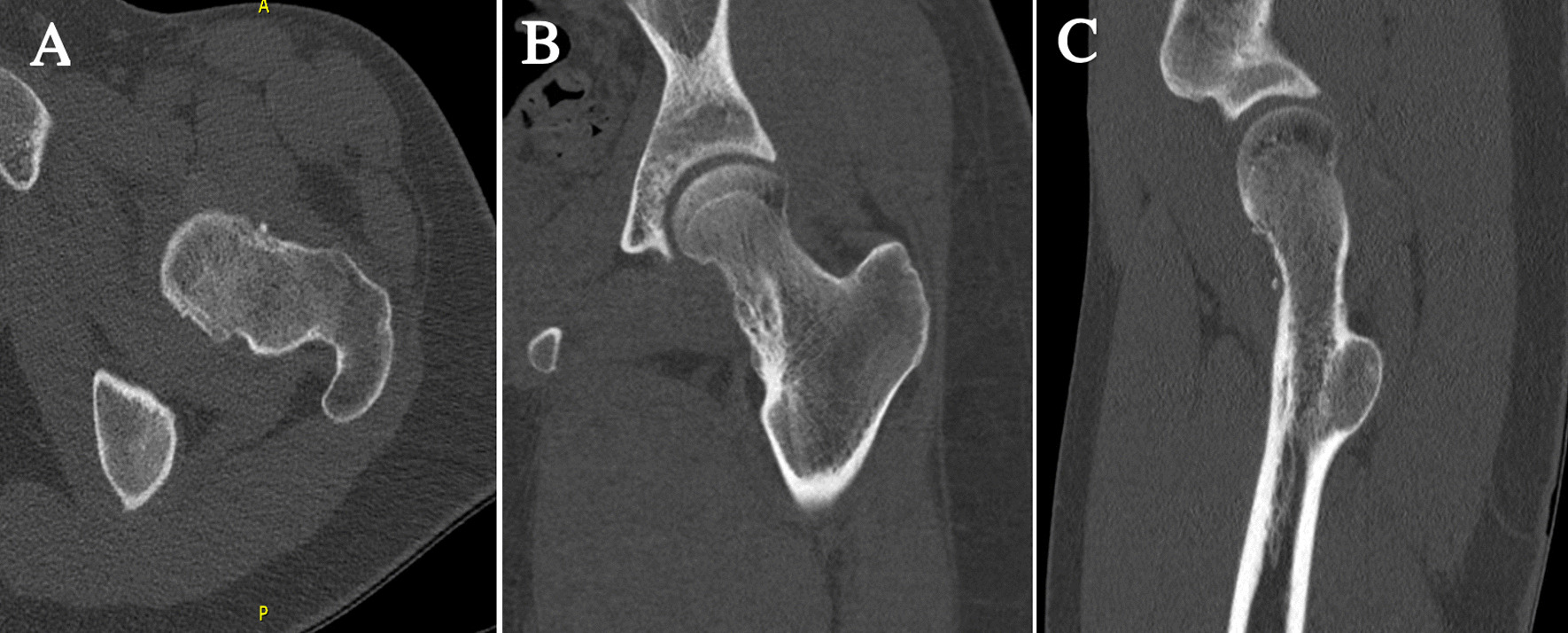
**A** Axial, **B** coronal, and **C** sagittal CT scans of the left hip at the 6-month follow-up visit showing complete excision with no recurrence

During his hospital stay, the patient was given cefuroxime 1.5 g intravenously as preoperative antibiotic prophylaxis and was kept on 750 mg every 8 hours for 24 hours postsurgery. He was kept on 1 L intravenous normal saline and was given intravenous paracetamol 1 g every 6 hours and intravenous push morphine 2 mg every 4 hours as needed for pain. He also required two doses of intravenous ondansetron 4 mg for nausea postsurgery. Postoperatively, the patient was allowed full-weight-bearing ambulation directly with hip range-of-motion exercise as no femoral compromise or internal fixation was necessary at the time of surgery. At 2 weeks postoperatively, he presented back to the clinic with an intact range of motion of the hip and good wound healing. Strengthening exercises were initiated. At 6 months follow-up, images revealed no occurrence, and he was able to return to his baseline physical activity (Fig. 7).

**Table:** 

Date	Event	Interventions
First week of December 2019	Left hip pain—clinic visit	Pain management + MRI and CT scan of the hip—diagnosis of hip osteochondroma
14 January 2020	Follow-up clinic visit—no improvement in pain	Surgical planning
29 January 2020	Surgery	Discharged home the next day after surgery
13 February 2020	Clinic follow-up	Good wound healing and hip range of motion
13 August 2020	Clinic follow-up	CT scan—no occurrence Return to baseline physical activity

## Discussion and conclusions

The following report is describing the case of a 14-year-old boy presenting for a limp secondary to two intra-articular hip osteochondroma lesions diagnosed on imaging. His symptoms were not responding to painkillers and interfering with his daily activity, which led to the decision to proceed with surgical excision. The traditional approach for these intraarticular lesions is either through an anterior or a posterior approach [6]. These approaches will include the dislocation of the femoral head to allow for full access to the hip joint. In this case, we decided to proceed with two incisions to address this tumor: a medial approach and an anterior approach allowing for the full excision of the tumor with no need for dislocation. This permitted the safe excision and minimized the risk of avascular necrosis of the femoral head associated with dislocation [7]. To our knowledge, this is the first case reporting excision of intra-articular osteochondroma utilizing two approaches. Given the benign nature of these lesions and their very low risk of malignancy transformation, they are managed conservatively [3]. No medical treatment has been described to date to tackle this pathology, and surgical intervention is reserved only for cases with refractory symptoms.

Osteochondromas are benign cartilaginous tumors in children [1]. There are only a few studies in the literature covering proximal femoral osteochondroma [5, 6]. Consequently, no consensus has been reached regarding the indications, outcomes, or optimal approaches for these lesions. Solitary lesions are diagnosed incidentally on imaging or by symptoms caused by the mass effect of the lesion [2]. No medical treatment is available for these lesions. It is generally recommended to observe osteochondroma in the pediatric population as few cases of regression have been reported and given the inherent risk of surgical intervention [8]. Surgery is considered only when symptoms caused by the mass effect of the tumor are interfering with daily life [3]. The primary purpose of this study was to describe the successful excision of an interscapular synovial hip osteochondroma using a dual surgical approach with no hip dislocation tailored to this patient.

The main concern with proximal femoral excision in the pediatric population is the risk of vascular compromise to the femoral head leading to osteonecrosis [7]. The femoral head receives its blood supply predominantly through the retinacular system of the medial femoral circumflex artery (MFCA) [9]. The MFCA is a branch of the profunda femoris artery and less frequently the common femoral artery [10]. It branches within the femoral triangle and crosses posteriorly behind the hip. This vascular system can be disturbed with deep dissection around the hip or dislocation to allow exposure [11]. The surgical dislocation of the hip has been described commonly to allow full exposure around the femoral head and acetabulum [12]. However, this technique, despite its excellent exposure, has been shown to cause disruption of the arterial supply to the femoral head and, hence, iatrogenic osteonecrosis [13]. For these reasons, it was essential in this case to provide the necessary exposure with a limited compromise to vascularity. This was further achieved by avoiding the need for hip dislocation.

The anterior approach to the hip was first described by Carl Hueter and later adopted by Smith Petersen [14]. It was initially adopted for the treatment of hip infections in children and then used for hip arthroplasty in adults. It is the only true internervous approach to the hip utilizing a muscle-splitting technique [15]. The plane between the sartorius and the TFL is accessed first and the deep plane between the rectus femoris and the gluteus medius later. It keeps the posterior soft tissue cover and hip adductors unharmed, all while preserving the blood supply to the femoral head [16]. This will allow for minimal blood loss, lower dislocation rates, earlier functional recovery, and better pain scores [17]. It provides good access to the anterior aspect of the hip with acceptable lateral and medial exposure as well; however, no posterior exposure is achieved [18]. Despite its numerous advantages, a few concerns remain regarding the anterior approach, especially the risk of injury to the lateral femoral cutaneous nerve (LFCN) [19]. It is most frequently a neuropraxia injury with limited long-term functional constraints. Wound hematomas and wound breakdown can occur in the early postoperative course due to the closure of the TFL sheath layer allowing for deep bleeders to reach the wound [20]. None of these complications was encountered in this case as careful dissection was done and thorough hemostasis and closure were achieved at the end of surgery.

The medial and posterior femoral neck are difficult areas to visualize through a traditional anterior approach and are probably not adequate for necessary access and instrumentation [5]. To allow access to the medial aspect of the hip, a second surgical incision was utilized with no need for hip dislocation. The medial approach to the hip was first described by Ludloff but later modified by several authors, including Ferguson [21, 22]. It was initially adopted as a surgical procedure for open reduction of developmental dysplasia of the hip [23]. The superficial intermuscular plane is first developed between the gracilis and the adductor longus, and then the deep plane is developed between the adductor brevis and magnus. This approach is carried out carefully while protecting the posterior division of the obturator nerve until the lesser trochanter is felt at the floor of the incision [21]. Due to the possible endangerment of the medial circumflex vessel, many surgeons shy away from this approach due to lack of familiarity with the exposure [24]. Therefore, careful dissection of the capsule with proper placement of the retractors is essential to avoid iatrogenic injury [21]. The advantages of this modified approach are not limited to visualization but also include simplicity, minimal dissection of soft tissue, direct access to structures, and limited blood loss.

The utilization of this dual approach allowed the patient to start early ambulation with no limitations in hip range of motion. At 2 weeks follow-up, the patient was able to ambulate with minimal pain, and strengthening exercises were started. To our knowledge, this is the first description of a resection of a symptomatic pediatric proximal femoral osteochondroma, using dual medial and anterior approaches. In the context of the recent available literature on the topic, our opinion is that this surgical exposure was optimal for both the safety and accessibility of this unusual condition.

## Data Availability

The data that support the findings of this study are available from the corresponding author upon reasonable request.

## Abbreviations

MRI: Magnetic resonance imaging
HME: Hereditary multiple exostosis
TFL: Tensor fascia lata
MFCA: Medial femoral circumflex artery
LFCN: Lateral femoral cutaneous nerve
